# Acceptability and Feasibility of Community Gardening Interventions for the Prevention of Non-Communicable Diseases among Indigenous Populations: A Scoping Review

**DOI:** 10.3390/nu15030791

**Published:** 2023-02-03

**Authors:** Rosana Emmanuel, Ursula M. Read, Antonio Jose Grande, Seeromanie Harding

**Affiliations:** 1 Department of Population Health Sciences, School of Life Course & Population Sciences, King’s College London, London SE1 1UL, UK; 2 Warwick Medical School, University of Warwick, Coventry CV4 7AL, UK; 3 Department of Medicine, Universidade Estadual de Mato Grosso do Sul—(UEMS), P.O. Box 351, Dourados 79804-970, Brazil

**Keywords:** Indigenous populations, indigenous people, nutrition, NCDs, community gardens, He Pikinga Waiora

## Abstract

Compared with non-Indigenous populations, Indigenous populations experience worse health across many outcomes, including non-communicable diseases, and they are three times more likely to live in extreme poverty. The objectives were to identify (1) the content, implementation, and duration of the intervention; (2) the evaluation designs used; (3) the outcomes reported; and (4) the enablers and the challenges. Using the PRISMA-ScR guidelines, a search of research databases and grey literature was conducted. Seven studies met the inclusion criteria. Papers reported on acceptability, nutrition knowledge, fruit and vegetable intake, self-efficacy, motivation, and preference concerning fruit and vegetable, diet, and gardening. No study measured all outcomes. All papers reported on acceptability, whether implicitly or explicitly. The evaluation used mostly pre- and post-intervention assessments. The effect of gardening on nutrition and gardening knowledge and fruit and vegetable intake was inconclusive, and was related to a general lack of robust evaluations. Applying the He Pikinga Waiora Framework, however, revealed strong evidence for community engagement, cultural centeredness, integrated knowledge translation and systems thinking in increasing the acceptability and feasibility of gardening in Indigenous communities. Despite environmental challenges, the evidence signaled that gardening was an acceptable intervention for the Indigenous communities.

## 1. Introduction

There are about 476 million Indigenous peoples globally, and they generally experience worse health and social outcomes than non-Indigenous populations [1]. Historic social and structural determinants drive these disparities. Indigenous people account for approximately 19% of the world’s poor and are three times as likely to live in extreme poverty as non-Indigenous people [1,2]. The COVID-19 pandemic amplified these inequities, with a general lack of adequate access to preventative care and management [3,4]. In 2016, Anderson et al. published an authoritative report comparing the health and social circumstances of 28 Indigenous and/or Tribal populations in 23 countries [5]. The magnitude and distribution of worse health outcomes varied across countries and among Indigenous groups within a geographical or political region [5]. Indigenous populations in Australia, Cameroon, Canada (First Nations and Inuit), Greenland, Kenya, New Zealand and Panama had a life expectancy at birth of >5 years lower than non-Indigenous populations [5], whilst those in China (Dai), Nigeria (Ijaw), Norway and Sweden had <2-year reduced life expectancy [5]. A high prevalence was reported for child malnutrition, child obesity and adult obesity. Adult obesity was prevalent in at least half of the Indigenous populations for which data were available [5]. Small-scale studies have also reported higher rates of non-communicable diseases (NCDs), such as diabetes, cardiovascular disease and hypertension [6]. For example, Pelletier et al. reported that in Canada, the prevalence of type 2 diabetes among ‘First Nations’ living on the reserve was three times higher than among the non-Indigenous population [6], and a report from the Australian government highlighted that 30% of Indigenous Australians aged 35–44 had high blood pressure, compared with 18% of non-Indigenous Australians in the same age group [7]. 

In 2011, the United Nations (UN) Permanent Forum on Indigenous Issues stated its commitment to implement culturally appropriate policies and actions for Indigenous peoples for NCD prevention [8]. In 2015, however, Indigenous peoples’ health received scant attention in the UN Sustainable Development Goals (SDGs) [9]. The seventeen goals aimed to end poverty and achieve sustainable development by 2030. They captured issues critical to equity, including well-being, gender equality, climate change and education. Indigenous people were mentioned six times—three times in the political declaration, one time under Goal 2 on Zero Hunger, one time under Goal 4 Quality Education and lastly in the section for follow-up and review at the national level [9]. Goals to address poverty alleviation and food insecurity are highly relevant to NCD prevention in Indigenous populations.

Globally, Indigenous peoples are at risk of food insecurity because of the degradation of their ecosystems [10,11]. Traditionally, Indigenous peoples have been farmers, hunters or pastoralists [1]. Several papers have reported on the lack of government support for protecting their ecosystems, inadequate access to nutritious foods, forced migration to urban areas in search of employment and loss of language and Indigenous knowledge over the generations [12,13,14,15]. Scholars have also emphasized the importance of food sovereignty, which extends beyond food security [16,17,18]. Food sovereignty relates to strengthening Indigenous communities and increasing social and environmental sustainability in producing, consuming, and distributing nutritious and culturally appropriate food [16,17,18]. Indigenous health researchers are increasingly focusing on sustainable subsistence food production, such as community gardening, to address the scarcity of nutritious foods and food sovereignty [12,14,15]. Additionally, Hond et al. reported that the Maori community gardens produce healthy food, support Indigenous peoples’ interests, self-determination and well-being and develop their ability to improve nutrition and health within their communities [19].

This review is partly guided by the He Pikinga Waiora (HPW) framework, which is particularly valuable for identifying potential enablers for sustainable and culturally appropriate interventions [20]. The HPW domains reflect best practices from the international literature: community engagement, cultural centeredness, integrated knowledge translation and systems thinking [20]. The framework emphasizes self-determination with the use of participatory research approaches to co-design projects with implementors and those who use or benefit from it [20]. It facilitates the translation and uptake of research into systemic practice, thereby increasing the potential of sustainability. 

The existing body of research on gardening interventions suggests that several challenges affect implementation and sustainability. Inadequate soil quality, water scarcity, access to/affordability of resources, availability of land, poor market linkages, lack of gardening knowledge, time constraints, high staff turnover, lack of support, labor burden and crop destruction by animals have been cited [21,22].There is inadequate mention of community garden interventions for Indigenous populations in published reviews [23,24,25,26] One review showed a positive impact on health and well-being for other vulnerable populations, including ethnic minorities and refugees, socioeconomically disadvantaged neighborhoods or low-income or food-insecure families [24]. No known existing or ongoing reviews of community gardening interventions in Indigenous populations were found through searches of MEDLINE, PROSPERO, COCHRANE or Joanna Briggs Institute (JBI) Evidence Synthesis.

It is important to understand how Indigenous contexts shape gardening interventions and whether this is a feasible approach to prevent the rising prevalence of NCDs [27]. A literature review on gardening interventions for NCDs or associated risk factors in Indigenous populations will give practical knowledge and an up-to-date understanding of the enablers and challenges to guide future research in community garden interventions for NCD prevention in Indigenous populations. This scoping review aimed to synthesize the literature on the acceptability and feasibility of community garden interventions to prevent NCDs or associated risk factors in Indigenous populations. Our research question addressed ‘What is the acceptability and feasibility of a community gardening intervention to prevent NCDs or associated risk factors in Indigenous populations?’ Our objectives were to identify (1) the content and mode of delivery of garden interventions, (2) the evaluation designs used, (3) the outcomes reported, (4) the enablers and (5) the challenges.

## 2. Materials and Methods

The scoping review used the framework developed by Arksey and O’Malley [28]. It was supplemented with the JBI methodological guidance document for scoping reviews, Preferred Reporting Items for Systematic Reviews, and Meta-Analysis extension for Scoping Reviews (PRISMA-ScR) [29,30]. Scoping reviews are beneficial when a body of literature has yet to be thoroughly reviewed or when it is complex or heterogeneous [28].The scoping review framework was based on the following steps: (1) identifying the research question; (2) identifying relevant studies; (3) carrying out study selection; (4) charting the data; (5) collating, summarizing and reporting the results. (6) Consultation. Stages 1–4 were conducted iteratively. Stage 5 was undertaken following stages 1–4, and stage 6 (consultation) occurred throughout the lifetime of the review between our research team and stakeholders. The initial research question that guided the systematic scoping review was: 

Can community gardens prevent NCDs among Indigenous populations?

### 2.1. Search Strategy 

#### 2.1.1. Search and Selection of Studies

An initial MEDLINE (Ovid) search was performed to identify text words in the title and any key terms that could be used as alternative search terms. Discussions among all authors resulted in the development of key terms. The subsequent search used Scopus, EMBASE, Global Health, Web of Science, *Indigenous Journal of Indigenous Health* and LILACS. Google Scholar, the World Bank, the United Nations, the World Health Organization, and Cultural Survival were searched for additional grey literature that could not be found through peer-reviewed databases. The search strategy is documented in Supplementary File S1. RE reviewed the reference lists of the identified studies for any further papers. Following a preliminary review of the studies, the following specific questions guided the charting of the information from the studies:

1. What was the content and mode of delivery of the community gardening programs?
2. What evaluation designs were used?
3. What outcomes were reported?
4. Using the HPW framework, what were the enabling factors?
5. What were the key challenges, and how were they resolved?

We applied the participants, concept and context (PCC) approach to frame key terms [29]. There is no universal definition of Indigenous and Tribal peoples. For this review, a variety of terms will be captured, including “Aboriginal,” “Tribe,” “Native,” “First Peoples/Nations,” “Māori,” “ethnic groups,” “Native Americans,” and “American Indians.” We are guided by the International Labour Organization Convention No. 169 criteria for identifying Indigenous peoples [31]: “people who speak unique languages, have unique knowledge systems, and hold invaluable knowledge of practices for the sustainable management of natural resources. They have a special relationship with and use their ancestral land. Their ancestral land is critical to their collective physical and cultural survival as people. Indigenous peoples have unique development concepts based on traditional values, visions, needs, and priorities*”* [31,32]. Following Kunpeuk et al., the concept of community gardens was defined as “shared spaces, public or private, where individuals or collective groups grow fresh fruits and vegetables” [33]. The context referred to community gardens in Indigenous territories worldwide. 

#### 2.1.2. Inclusion and Exclusion Criteria

Studies included met the following criteria: (1) community garden interventions aimed at preventing NCDs or associated risk factors among Indigenous populations; (2) implementation and evaluation of community garden interventions; (3) reported in English in peer-reviewed or grey literature; (4) published before the end of May 2022. Studies excluded were (1) community garden studies with non-Indigenous populations; (2) community garden interventions unrelated to NCDs or associated risk factors; (3) studies focusing on community gardens with no implemented gardens; (4) reported in languages other than English; (5) study protocols. RE screened the retrieved abstracts and removed duplicates. Conflicts were discussed with co-author SH and resolved through consensus. Complete papers were retrieved if abstracts met the eligibility criteria. 

### 2.2. Charting, Collating and Summarizing the Data 

We used Microsoft Excel to create a data extraction template that could be used to chart the data. Topics covered were based on the research questions including (i) study characteristics: author, year of publication, country, objective, setting, sample size; (ii) intervention content; (iii) evaluation design; (v) enablers using the HPW framework; and (vi) challenges. The HPW framework captures information on four domains: (i) ‘cultural centeredness’ refers to community ‘voice’ for problems and solutions, reflexivity and structures and resources for the successful implementation of effective interventions to achieve health equity; (ii) community engagement emphasises partnership and co-development with community members and power-sharing; (iii) systems thinking refersto the importance of considering the implementation of interventions from a range of perspectives, levels and understandings, with an emphasis on holism and connection among levels, institutions, systems and people; (iv) integrated knowledge translation refers to co-innovation involving knowledge users as equal partners alongside researchers, policymakers and practitioners to develop relevant and valuable perspectives for end users. For each selected paper, each domain was rated “high”, “medium,” “low,” or “negative” [20]. The process of data charting was iterative, and the form was revised following team discussions on the papers. We used the evidence tables to organize and synthesize the data to produce a narrative addressing the questions outlined above. 

### 2.3. Stakeholders’ Consultation 

The review was prompted by discussions with our Indigenous stakeholders in our studies on NCD prevention in Latin America and the Caribbean [34]. Stakeholders included Indigenous university students, health professionals, church leaders and congregants. During concept mapping workshops, they reported on the impact of their ecosystem’s degradation on their food systems, lack of access to healthful foods, and a need to support gardening interventions [35]. 

## 3. Results

**Figure 1:**
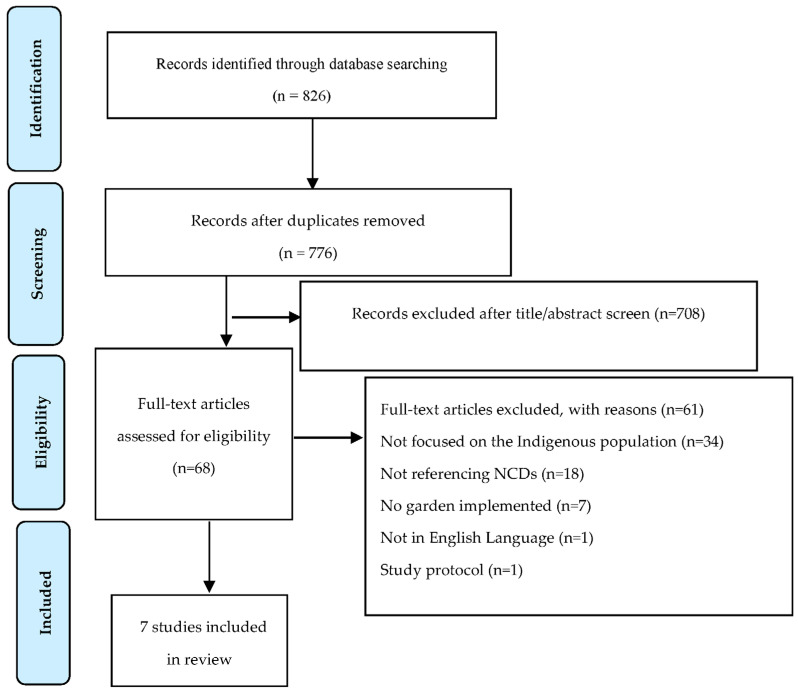
PRISMA diagram: (adapted from Moher et al. [43].

Figure 1 shows the selection process of studies included in this scoping review. Seven studies were considered eligible from the initial eight hundred and twenty-six studies retrieved. One was published in 2006 [36], five between 2014 and 2020 [37,38,39,40,41] and one in 2021 [42]. Two studies were conducted in Australia [36,37], two in Canada [38,39] and three in the United States of America (USA) [40,41,42]. Sixty-one papers were excluded. Thirty-four did not focus on Indigenous populations, eighteen did not relate to NCDs and/or risk factors, seven did not report on an implemented garden, one was in a language other than English and one was a protocol paper (authors contacted but findings not ready for sharing). 

### 3.1. Summary Characteristics 

Table 1 summarizes the setting, sample size, objectives, intervention content, delivery and duration, and outcomes extracted from each study.

#### Setting, Sample Size and Objectives

Four studies used gardens in school settings, and three used gardens in public settings. In total, the studies enrolled 616 participants; three reported a sample size of <50 participants [37,40,41], one reported a sample size of 50–99 participants [36] and three reported 100–200 participants [38,39,42]. Two school studies were in Australia, and two were in Canada. Viola et al. explored the effectiveness of school gardens as a nutritional educational tool [36], and Hume et al. explored the acceptability of a gardening program to promote a range of nutrition and gardening activities in remote schools without on-site horticultural support [37]. Triador et al. explored fruit and vegetable preferences and home consumption among First Nations schoolchildren in Canada [38]. Hanbazaza et al. assessed changes in First Nations schoolchildren′s knowledge, preferences and home consumption of fruit and vegetables [39]. All three public garden interventions were in the USA. Brown et al. explored the feasibility of a group gardening intervention and the potential for collecting health outcomes [40]. Johnson-Jennings et al. explored the feasibility of gardening as an obesity intervention among Indigenous children and families in a Northern Midwestern urban community at risk of homelessness [41]. Lombard et al. explored whether gardening could promote healthy eating habits in the Navajo Nation [42].

### 3.2. Content, Delivery and Duration

School garden interventions included gardening, nutrition knowledge and/or activities and preparation of nutritious snacks/lunch [36,37,38,39]. Gardens were outside the classrooms [36,37,39] and used portable beds [37] or earth box gardens in classrooms, the library [38,39] or in the community [39]. In one study, formal nutrition and gardening education lessons were integrated into the core curriculum in primary and secondary schools and delivered by teachers [36]. In the other three studies, teachers were given the resources and decided how to deliver the intervention [37,38,39]. One study appointed coordinators for the project to assist with facilitation at the local level [36], and three used Indigenous Elders to assist with intervention delivery [36,38,39]. The intervention sessions were either daily [36] or weekly [37,38,39], and the duration of the intervention ranged from 4 months [37] to 24 months [39]. 

Public garden interventions included gardening, nutrition knowledge and/or activities, preparation and/or tasting of nutritious meals and taking the produce home for consumption with the family [40,41,42]. Two used raised garden beds [40,42], and one used a rooftop garden [41]. Indigenous professionals (e.g., tribal agriculture extension agents, staff members, local master gardener) developed a series of ten structured gardening and food-related sessions [40], and Indigenous Elders provided mentorship [41]. Intervention sessions varied from several times per week [41] to weekly [40] or weekly and monthly [42].

### 3.3. Outcomes

Table 1 summarizes the findings on acceptability, nutrition-related knowledge, fruit and vegetable intake, fruit and vegetable self-efficacy, motivation or preference, dietary self-efficacy, gardening-related knowledge and gardening self-efficacy or motivation.

#### 3.3.1. School Garden Outcomes

School garden interventions: Acceptability of the intervention was apparent in all studies [36,37,38,39], though not explicitly reported by three studies [36,38,39]. In Hume et al. study, the teachers found the classroom resource a beneficial planning and time-saving tool [37]. In the other three studies, students reported enjoying working outside the classroom in a less structured and more practical environment [36], and retention rates were high for elementary school students [36,38,39]. Only three school garden intervention studies explicitly reported nutrition-related knowledge [36,37,39]. Viola et al. reported an increase in nutrition knowledge among elementary school students but a decrease among secondary school students, with no statistical significance [14]. Hume et al. reported an increase in nutrition-related classroom activities [37]. In Hanbazaza et al. study, children increased their knowledge at the end of the intervention [39]. Fruit and vegetable intake was reported by two studies. Triador et al. reported a non-significant increase in consumption at home of ten of seventeen fruits and vegetables [38]. Hanbazaza et al. reported no statistically significant change in the home food consumption of any fruit or vegetable over the intervention [39]. Two studies reported total preference scores for fruit and vegetables. Triador et al., reported that children’s vegetable preference scores improved over seven months; only tomatoes, however, showed statistically significant improvements [38]. Hanbazaza et al. reported statistical significance for fruit preference [39]. Viola et al. reported that gardening knowledge and skills increased over the six-month intervention period [36]. In the study by Hume et al., teachers reported a positive increase in the hours spent gardening and in gardening-related classroom activities [37]. None of the school garden interventions reported on gardening self-efficacy or motivation. 

#### 3.3.2. Public Garden Outcomes

As with school gardens, the acceptability of public gardens was also apparent, though again not explicitly stated. For example, gardens were perceived as culturally relevant and feasible [41]. Although two studies reported that retention rates were low [40,42], participants considered community gardens a positive healthy activity that promoted community relations [40]. Community gardens also diffused into home gardens [42]. Two public garden interventions explicitly addressed nutrition-related knowledge. Johnson-Jennings et al. reported that participants endorsed healthier food perceptions [41]. Lombard et al. reported a non-significant decrease in fruit and vegetable preparation knowledge [42]. Brown et al. [40] and Lombard et al. [42] reported no change in fruit and vegetable consumption. Change in intake was not reported in the study by Johnson-Jennings et al.; however, vegetables were shared among all community members and used for weekly meals [41]. Three studies reported on self-efficacy and motivations to eat fruit and vegetables. They did not show any significant changes, but a high level of motivation was noted by Brown et al. [40], and a non-significant increase in confidence in consuming fruits and vegetables daily was reported by Lombard et al. [42]. All public garden intervention studies reported on gardening knowledge. Brown et al. reported a perceived lack of gardening knowledge [40], and Johnson-Jennings et al. reported an increase in traditional ecological knowledge (e.g., Elders were natural role models who shared traditional ecological knowledge and increased community awareness) [41]. Lombard et al. [42] reported a non-significant increase in gardening knowledge. Two studies reported on gardening self-efficacy. Brown et al. reported that garden stages of change scores improved among the intervention group compared with the comparison group [40]. Lombard et al. reported a non-significant increase in gardening self-efficacy [42]. Brown et al. found no significant change in physiological (glycosylated hemoglobin, body mass index, systolic blood pressure) or mental health measures [40].

### 3.4. Evaluation Designs

Table 2 summarizes the evaluation designs. All four school garden interventions used a pre–post design without comparator schools, three used mixed methods [36,37,39] and one study used only a quantitative survey at the end of the intervention [38]. Quantitative assessments included the healthy food pyramid activity to assess the nutrition knowledge of students [36], teacher survey to assess student time spent in cooking and gardening [37] and assessments of preferences and self-reported home consumption of fruits and vegetables [38,39]. In other studies, nutritional knowledge was assessed using a drawing of a healthy dinner plate [36], time spent in nutrition-related classroom activities [37], and by children writing about vegetables [39]. One school garden intervention assessed teacher acceptability and the monetary cost of the intervention using open-ended questions [37]. Researchers used a reflective notebook and events journal to record nutrition-related activities [36] and a horticultural support register to record researchers’ and teachers’ email and phone exchanges [37].

Evaluation of the three public garden interventions used a randomized control design [40] and a pre–post design without comparator gardens [41,42]. In addition to survey measures such as fruit and vegetable intake and fruit and vegetable self-efficacy, motivations or preferences, these studies also used surveys to assess nutrition-related knowledge/activities [41,42], gardening knowledge [40,41,42] and gardening self-efficacy or motivation [40,42]. Psychological and physiological assessments were included in the study by Brown et al. [40]. They used the Profile of Mood States Inventory, World Health Organization Quality of Life, and collected self-reported data on body mass index, blood pressure and glycated hemoglobin [40]. Qualitative interviews explored the influences that promote or hinder community gardens and the use of locally grown foods [40], traditional ecological knowledge [41] and how best to promote gardening among community members [42]. 

### 3.5. Enablers Using the HPW Framework 

Detailed information for enablers for each of the four HPW domains is provided in Supplementary File S2. In general, only one study, the school garden study by Hume et al. [37], was classified as low for most domains. We aggregated the domains due to overlapping evidence supporting the domains. 

#### 3.5.1. Cultural Centeredness and Community Engagement

Six of the seven studies were classified as having integrated a high level of cultural centeredness and community engagement [36,38,39,40,41,42]. This was evident from co-development with community advisory groups [36], research steering committees [38,39] and community advisory boards [40,41]. These included tribal Elders and Indigenous stakeholders (health workers, school principals, staff agriculture education specialists, master gardeners). The prior existing relationship between researchers and the community was evident in three studies [36,41,42]. Six of the seven studies were also classified as having high levels of reflexivity. Evidence related to researchers and stakeholders adapting the intervention to align with the community’s needs [36,38,39,40,41,42]. For example, in the study by Lombard et al. with the Navajo community, the frequency of the workshops was reduced from weekly to monthly to meet family and work obligations [42]. Brown et al. provided strong evidence of leveraging partnerships to access resources for the community. On completion of the study, the Tribal government allocated ~40 acres of prime agricultural land for community members to grow fruit and vegetables [40]. Evidence of community engagement related to establishing links with Elders or community members who had an existing garden or who wanted to start a garden [36], using community-based participatory research methods [38,39,40,41], had longstanding relationships with the community [36,41,42], selling of excess vegetables in the market [41], organizing a harvest festival [42], sharing harvested food [39,40,41,42] and engaging local Indigenous expertise [36,40,41].

#### 3.5.2. Systems Thinking and Integrated Knowledge Translation

Most of the studies showed high levels of holistic and systems thinking with the incorporation of the perspectives and skills of stakeholders from the community, schools or health professionals [36,38,39,40,41,42]. For example, the study by Viola reported on community relationships that allowed for feedback on data-gathering techniques before implementation and connections with Elders to access and leverage existing community resources [36]. Three studies targeted different ecological levels [40,41,42]. Johnson-Jennings et al. ensured the garden was open to all community and family members, and vegetables were shared among all community members and used for weekly meals [41]. Four studies focused on change at the school and student levels [36,37,38,39]. Six studies were classified as having high knowledge translations [36,38,39,40,41,42]. These garden interventions integrated the Indigenous knowledge from community leaders, Elders, Tribal health workers, schoolteachers, gardeners, agricultural education workers and other essential stakeholders.

## 4. Challenges for Community Gardening Interventions

Water scarcity was a challenge for schools and public gardens [38,40]. In the study by Brown et al., community members learned how to install drip irrigation systems so that raised beds were irrigated with rainwater from catchment cisterns [40]. Challenges for school garden interventions related to high school absenteeism, teaching staff turnover [36], perceived risk of vandalism (though actual vandalism was not reported) [37] and limited availability of nutritious foods [39]. Many more challenges were cited for public gardens—lack of confidence in collective decision making, lack of community experts in gardening, poor soil quality, limited access to public transportation, the convenience of buying cheap processed food [40], community transience [41] and financial barriers [42]. 

## 5. Discussion

This scoping review aimed to systematically identify the literature on community garden studies to prevent NCDs and related risk factors in Indigenous populations. Seven of the eight hundred and twenty-six studies retrieved were included in this synthesis, four of which used school gardens and three used public gardens. They broadly aimed to explore if a gardening intervention could improve nutrition and gardening-related knowledge, skills and practices and fruit and vegetable preferences and intake. Intervention content spanned a mixture of education and interactive gardening sessions that were mainly delivered by teachers and Indigenous staff. Evaluation used mainly pre- and post-intervention assessments. The influence of gardening on nutrition and gardening-related outcomes and on fruit and vegetable intake was inconclusive, and was related to a general lack of robust evaluations. One study reported on other risk factors such as obesity, diabetes, hypertension and mental health, and it showed no impact. Positive evidence for the HPW domains, however, strongly signaled that gardening was an acceptable intervention for the Indigenous communities despite environmental challenges. 

A comparison with the findings of gardening interventions among non-Indigenous populations highlights some distinct commonalities and differences, particularly in designs. Since 2015, sixteen reviews have assessed the impact of gardening on physical and mental health and well-being. These reviews captured four hundred and forty-four studies in twenty-six countries which reflected a strong growing global interest in gardening as a public health intervention. The reviews targeted cardiovascular [44], chronic illnesses [23,45,46,47,48] and related risk factors [21,23,24,25,26,33,44,45,48,49,50]. Four reviews focused on school gardening studies [22,25,51,52]. Only four studies of Indigenous gardens were included in the sixteen reviews, two of which are included in our review [36,40]. Generally, these reviews show a pattern of positive but weak associations between gardening and improvement in dietary outcomes, including fruit and vegetable intake, and in physical and mental health. Two reviews included a meta-analysis. Kunpeuk et al. showed a positive association with a reduction in body mass index [33], whilst Qi et al. indicated an increase in children’s fruits and vegetables and in preferences, but no effect on body mass index or waist circumference [50]. 

A common feature of both our review and those of gardening interventions with non-Indigenous communities was a general lack of robust evaluations. Non-randomized designs and pre–post-test designs were commonly used to evaluate outcomes [45]. Qualitative evaluations were sometimes used to strengthen the interpretation of findings [22,24,44,46,49,51,52]. Within an evidence-based framework for evaluating the effectiveness of public health interventions, randomized designs are recognized to be problematic particularly in the context of implementation in community settings. Contamination, that is, participants from control areas visiting intervention areas and vice versa, can confound outcomes. Gardening interventions are complex interventions, and it is critical to understand how the complexity of context affects the mechanisms of change and thus the outcomes. Evaluation of complex intervention studies often uses non-randomized designs with comparator arms, matched for key confounders (e.g., socio-demographic composition) with qualitative evaluation [53]. In our review, only one study included a comparator arm. It is important to emphasise that non-experimental participatory action research is particularly appropriate for Indigenous communities not only because of the geographical remoteness of some communities but also because it engenders academic–community trust [54]. In addition to issues relating to evaluation designs, there was also inadequate reporting of retention rates, intervention delivery (e.g., fidelity to protocols) and outcomes (gardening or nutrition knowledge, fruit and vegetable intake). Lombard et al. was the only study that reported consistently across acceptability of the intervention, nutrition and gardening-related knowledge and activities and fruit and vegetable intake [42]. 

A strength of the studies we reviewed related to the detailed considerations of community engagement, and this provided important insights about the enablers and challenges. There was a strong emphasis in the studies on partnerships and co-development with communities, to align the intervention with their needs. Though the HPW framework was not used in any of the published reviews, our findings resonate partially with those identified by Burt et al. [49]. Their review of thirty-one studies explored the social benefits of gardening and concluded that community garden interventions create community engagement by facilitating more social connection, community sharing and neighborhood participation [49]. There was sparse mention of community engagement in the other fifteen reviews of non-Indigenous gardening studies [24,25,44]. Though designed for implementation studies with Indigenous communities, it is easy to see the value of the HPW framework for evaluations of gardening interventions. In the current context of climate change impacts on the sustainability of food systems and on food justice [22,49], the emphasis on systems thinking, community engagement and integrated knowledge translation is pertinent.

Our review identified several challenges for Indigenous community gardens that correspond with those for non-Indigenous community gardens. These included poor soil quality, water stress, affordability of inputs, availability of land, time constraints, lack of gardening knowledge, high staff turnover rates and lack of support for sustainability [21,22]. 

## 6. Limitations of the Review

The inclusion and exclusion criteria may have reduced the number of studies. The review was limited to studies reported in English. The search criteria may have missed publications that identified Indigenous groups using other phrases, such as ‘nomads’. Some studies may not have used the term ‘Indigenous’ but referred to geographical areas where Indigenous tribes inhabit. The modest number of studies demonstrated a limited evidence base and the need for further research given its potential public health value for NCD prevention.

## 7. Conclusions

Indigenous peoples face significant inequities, including poverty, access to care and availability of nutritious foods, which impact their health and well-being. Although there was limited evidence on the prevention of NCDs, there was consistent evidence for the acceptability of gardening interventions in Indigenous communities and for the enablers and challenges. The evaluations were generally limited and focused on fruit and vegetable intake. Only one study reported on other outcomes such as diabetes, hypertension or BMI. There was, however, strong evidence for cultural centeredness and community participation to promote the acceptability and feasibility of community gardens for NCD prevention in Indigenous communities. Overall, this review provides important insights into the enablers and challenges around community garden interventions for the prevention of NCDs and associated risk factors among Indigenous communities, and guidance to drive future research in this area.

## Figures and Tables

**Table 1:** Summary Characteristics of Scoped Studies.

Author(s), Year, Country	Setting	Sample Size	Objective	Content, Delivery and Duration	Outcomes
Viola (2006), [36] Australia	School	86 children Secondary students (SS) (*n* = 66) F = 37, M = 29 Primary students (PS) (*n* = 20) F = 8, M = 12	Examine the effectiveness of school gardens as a nutritional education tool in Indigenous Australian school settings	Formal nutrition and gardening education lessons; prepared nutritious meals; delivered by teachers daily; coordinators and Indigenous Elders assisted; gardens were outside of the classroom	Acceptability (SS = 23%; PS = 100%) Nutrition knowledge (SS↓ 73.7–56.6%; PS↑ 52.6–66.2%; statistical significance not reported), Gardening knowledge/skills (↑ perceptions, but no measure reported)
Hume et al., 2014, [37] Australia	School	48 children 8–14 years	Determine the feasibility of a low-cost program to get remote schools started in gardening and nutrition activities	Nutrition, garden, and cooking activities in the classroom; horticultural support; garden beds installed; weekly sessions; delivered by teachers 4 months intervention	Acceptability (resource helped teachers plan and save time) Nutrition-related activities (↑ 1.0–1.6 h, statistical significance not reported), gardening-related activities (↑ 0.5–2.0 h, statistical significance not reported)
Triador et al., 2015, [38] Canada	School	116 children 9.0 ± 1.8 years M = 65%, F = 35%	Evaluate changes in children’s self-reported preferences for vegetables and fruit in response to the school gardening intervention and children’s self-reported home consumption	Earth box kids garden education paired with a weekly snack program; weekly delivery by teachers; supported by community Elder; Earth box gardens were in classroom and library; 7 months intervention	Acceptability (65% retention rate) Fruit and vegetable intake (↑ 59% self-reported increase 10/17 fruits and vegetable, not statistical significance), fruit and vegetable preference (↑ 81–83%, statistical significance only for tomatoes)
Hanbazaza et al., 2015, [39] Canada	School	116 children 9.0 ± 1.8 years 9.8 ± 1.7 years	Assess changes in First Nations schoolchildren′s knowledge, preferences, and their home consumption of fruits and vegetables	Earth box kids garden education intervention included a 4-month weekly snack program; supported by community Elder; Earth box gardens were in classrooms/library/community; weekly delivery by teachers; 18 months intervention	Acceptability (56.8% retention rate) Nutrition knowledge (↑ 4.5 ± 1.0–4.9 ± 0.1 0.005, *p* = 0.0005, statistical significance) Fruit and vegetable intake (no significant changes in home fruit and vegetable consumption), fruit and vegetable preference (fruit preference) 41.1 ± 4.3 vs 42.7 ± 3.0, *p* = 0.003, statistical significance)
Brown et al., 2020, [40] USA	Public	41 Native Americans 21 years +	Determine the feasibility of a group gardening program and the potential for collecting health outcomes	Raised garden beds; 10–90-min structured sessions with hands-on gardening and food preparation activities delivered weekly/bi-monthly; developed by Indigenous professionals; vegetables shared with participants; 7 months intervention	Acceptability (90% retention rate), Fruit and vegetable intake (No change), fruit and vegetable motivation (90%), gardening knowledge (Perceived lack), gardening motivation (*p* = 0.111; non-significant improvement) Physiological, psychological (non-significant change)
Johnson-Jennings et al., 2020, [41] USA	Public	169 Navajo adults 18+ years of age or older	Determine the feasibility of a group gardening program and the potential for collecting health outcomes	Nutrition education and culturally engaged physical health promotion; rooftop garden; preparing nutritious meals; vegetables shared among community members and used for weekly meals; Indigenous Elders provided mentorship; delivery 3–4 times per week; 12 months intervention	Acceptability (perception of cultural viable and excitement for participation), nutrition knowledge (improved healthier food perceptions, statistical significance not reported), dietary self-efficacy (*p* = 0.450, non-significant change), gardening knowledge (↑ perceived increase)
Lombard et al. (2021). USA	Public	169 Navajo adults 18+ years of age or older	Assess a theory-based and culturally relevant community garden intervention to promote adult gardening and fruit and vegetable consumption in two Navajo communities.	Outdoor raised garden beds; gardening and healthy eating adult education; promoting positive social norms around gardening through community outreach activities; delivery weekly/bi-monthly/monthly 12 months intervention	Acceptability (community gardens diffuse into some home gardens), nutrition knowledge, fruit and vegetable intake (no significant differences intake in daily serving), fruit and vegetable self-efficacy (↑ 63–72%, *p* = 0.21, non-significant increase), garden self-efficacy (↑ 50–53%, *p* = 0.78, non-significant increase)

**Table 2:** Evaluation designs.

Author(s) and Country	Evaluation Design	Data Collection Method
Viola A. 2006, [36] Australia	Pretest–post-test design	Semi-structured interviews; reflective journal; curriculum matrix; event log; activity sheets (‘My Healthy Dinner Plate Activity’ and ‘The Pyramid Activity’)
Hume et al., 2014, [37] Australia	Pretest–post-test design	Teacher surveys with open-ended questions on acceptability and gardening success; horticultural support register
Triador at al., 2015, [38] Canada	Pretest–post-test design	Vegetable Preference Adapted Surveys administered in the classroom
Hanbazaza et al., 2015, [39] Canada	Pretest–post-test design	Fruit and vegetable Adapted Questionnaire administered; written responses (children’s knowledge of fruit and vegetables was assessed by asking children to write down 5 fruits and vegetables that they knew)
Brown et al., 2020, [40] USA	RCT (1) Group-based Community gardening program (2) control (no gardening)	Self-reported surveys: Profile of Mood States Inventory; a modified version of the Center for Epidemiological Studies—Depression Scale; World Health Organization Quality of Life Questionnaire—BREF (WHOQOL-BREF), Stages of Change Scale
Johnson-Jennings et al., 2020, [41] USA	Pretest–post-test design	Surveys (dietary self-efficacy survey); focus groups on research designs and curriculum; interviews on effectiveness of gardening project, on healthy eating /exercise, and what worked well in the intervention
Lombard et al., 2021, [42] USA	Pretest–post-test design	Food frequency fruit and vegetable questionnaire administered; interviews; workshops

## Data Availability

All data used in this review are available in cited published literature.

## Supplementary Materials

The following supporting information can be downloaded at: https://www.mdpi.com/article/10.3390/nu15030791/s1, Search Terms, Supplementary File S1; and Enablers using the HPW Framework Supplementary File S2.
